# Ageism and Associated Factors in Healthcare Workers: A Systematic Review

**DOI:** 10.3390/nursrep14040295

**Published:** 2024-12-16

**Authors:** Laura Fernández-Puerta, Alexis Caballero-Bonafé, Juan Ramón de-Moya-Romero, Antonio Martínez-Sabater, Raquel Valera-Lloris

**Affiliations:** 1 Valencia Clinic Hospital, 46010 Valencia, Spain; caballero_alebon@gva.es (A.C.-B.); demoya_jua@gva.es (J.R.d.-M.-R.); antonio.martinez-sabater@uv.es (A.M.-S.); valera_raq@gva.es (R.V.-L.); 2 Nursing Department, Faculty of Health Sciences, European University of Valencia, 46010 Valencia, Spain; 3 Care Research Group (INCLIVA), Valencia Clinic Hospital, 46010 Valencia, Spain; 4 Nursing Department, Faculty of Nursing and Podiatry, University of Valencia, 46010 Valencia, Spain; 5 Nursing Care and Education Research Group (GRIECE), Nursing Department, Faculty of Nursing and Podiatry, University of Valencia, 46010 Valencia, Spain

**Keywords:** ageism, healthcare workers, geriatric care, geriatric education

## Abstract

**Background**: Ageism refers to the presence of stereotypes, prejudices, and discrimination against older adults based on their age. In healthcare settings it negatively impacts opportunities for treatment, rehabilitation, and cure opportunities. This study aims to assess the presence of ageism among healthcare workers toward older patients and to identify the associated sociodemographic, personal, and work-related factors. **Methods**: A systematic review of the literature was performed using PubMed, Embase, CINAHL, and Scopus. Studies that assessed the presence of ageism among healthcare professionals through a quantitative or mixed methodology and published between 2014 and 2024 were included. **Results**: Fifteen articles met the inclusion criteria. Healthcare workers generally exhibited low rates of ageism; however, results varied across studies. Although the available literature is limited, workers with less knowledge about aging and less experience, especially in geriatric units, showed higher ageism scores. Intergenerational contact and a wish to work with older people appeared to be important factors for promoting a positive relationship with older adults. Other sociodemographic and sociocultural factors, such as age and sex, were not related to ageism. Workload and work-related factors, such as stress or lack of personnel, might be associated with ageism, but few studies were found to be available to confirm these results. **Conclusions**: Ageism scores among professionals were low. Gerontological education and clinical and family experience could help reduce ageist attitudes toward older patients among health professionals.

## 1. Introduction

It is undeniable that the aging of the global population has become a major public health concern in the 21st century. Projections indicate that the number of people over 60 is expected to double, while the number of individuals aged 80 or older is projected to triple between 2020 and 2050 [1]. For the prosperity of the global population and its adaptation to the ongoing epidemiological transition, geriatric issues must be considered of vital priority. However, older adults continue to face discriminatory and stereotypical attitudes, both explicitly and implicitly. The interactions they experience with these stereotypes, prejudices, and discrimination are commonly referred to collectively as ageism [2]. Ageism is fundamentally based on issues related to cognitive, affective, and behavioral perspectives [3]. The term “ageism” was first used by Butler, (1969) [4] defining it as an ideology and a process that could evolve into discrimination against the elderly due to their advanced age, similar to the prejudices and stereotypes inherent in racism and sexism.

In Western countries, aging is associated with beliefs of frailty, weakness, dependency, and unproductivity, among others. Moreover, health issues in older adults are naturalized and considered inherent to the aging process [5]. Consequently, numerous studies have been conducted in recent years to identify the determinants related to the presence of ageism. A systematic review conducted by Marques et al. [2] found that individual characteristics, such as anxiety about aging or fear of death, are associated with more ageist attitudes. Similarly, other individual determinants, such as being younger, male, and other sociodemographic characteristics, like lower educational level, low socioeconomic status, and single civil status, have proven inconclusive in determining their influence on ageism, although numerous studies associate their presence with more negative attitudes towards old age [2]. In terms of the cultural context, there is evidence that having a high proportion of older people in the environment acts as a protective factor against ageism [6]. Cultural origin, ethnicity, and religiosity could similarly be linked to less ageist attitudes, especially in collectivist countries where older people are respected and revered [7].

Just as in the general population, discrimination against older adults is also present in healthcare, becoming particularly evident during the COVID-19 situation [8]. In this context, the effects of ageism experienced by older adults are well documented, leading to restricted access for them and their families to various existing resources, and reducing opportunities for treatment, rehabilitation, and cure. This impacts the quality of life and well-being of older adults, exposing them to worse health conditions, reduced longevity, health risk behaviors, poor social relationships, physical and mental illnesses, and cognitive decline [9,10]. Moreover, this also leads to self-stigmatization in these patients [11].

Therefore, various studies have been conducted to understand the presence of ageist attitudes among healthcare professionals when caring for older adults. The results have shown a range of attitudes, from negative [12,13] to slightly positive, [14] and to positive [15,16]. Additionally, these studies found that factors such as insufficient training in aging and lower educational levels [17], as well as erroneous beliefs—like the perception that older adults are less competent and more difficult to treat [18]—are associated with age discrimination in healthcare. Indeed, several studies report that healthcare workers prefer to work with younger patients [19]. However, most studies conducted to date have focused primarily on nurses [19]. To our knowledge, only one qualitative systematic review has explored ageism among all healthcare professionals. This review revealed that doctors perceive the care of older adults as complex and dependent, while nurses described it as a significant workload [20]. Nevertheless, no review has described the current state of ageism among all healthcare professionals from a quantitative perspective, using validated scales that comprehensively address all aspects of ageism (negative attitudes, prejudices, and discrimination). Furthermore, to date, there is a lack of systematic knowledge about which personal and job-related factors are associated with higher levels of ageism among different healthcare professionals toward older patients. A more precise understanding of these factors may facilitate the development of targeted interventions to address them, thereby ensuring quality, inclusive, and respectful care, with the ultimate goal of enhancing the well-being of older adults and promoting more positive attitudes toward them.

## 2. Aims

The primary aim was to assess the presence of ageism among healthcare workers toward older patients and to identify the sociodemographic, personal, and work-related factors associated with the presence of ageism.

## 3. Methodology

This study was guided by the Preferred Reporting Items for Systematic Reviews and Meta-Analyses (PRISMA) [21]. This protocol was published in the International Prospective Register of Systematic Reviews (PROSPERO) with the ID number CRD42024548783. Studies describing discrimination against the elderly population in healthcare settings were included, according to the following inclusion and exclusion criteria:

(i) Studies assessing the presence of ageism among different healthcare professionals toward older patients; (ii) primary studies; (iii) quantitative or mixed-method studies; (iv) studies published between 2014 and 2024; and (v) studies in English or Spanish. Exclusion criteria were (i) studies that included health sciences students or faculty members; (ii) studies with a qualitative methodology; and (iii) studies of low quality when assessed by the Joanna Briggs Institute Critical Appraisal Tools checklist (JBI) for cross-sectional studies.

### 3.1. Search Strategy

**Figure 1:**
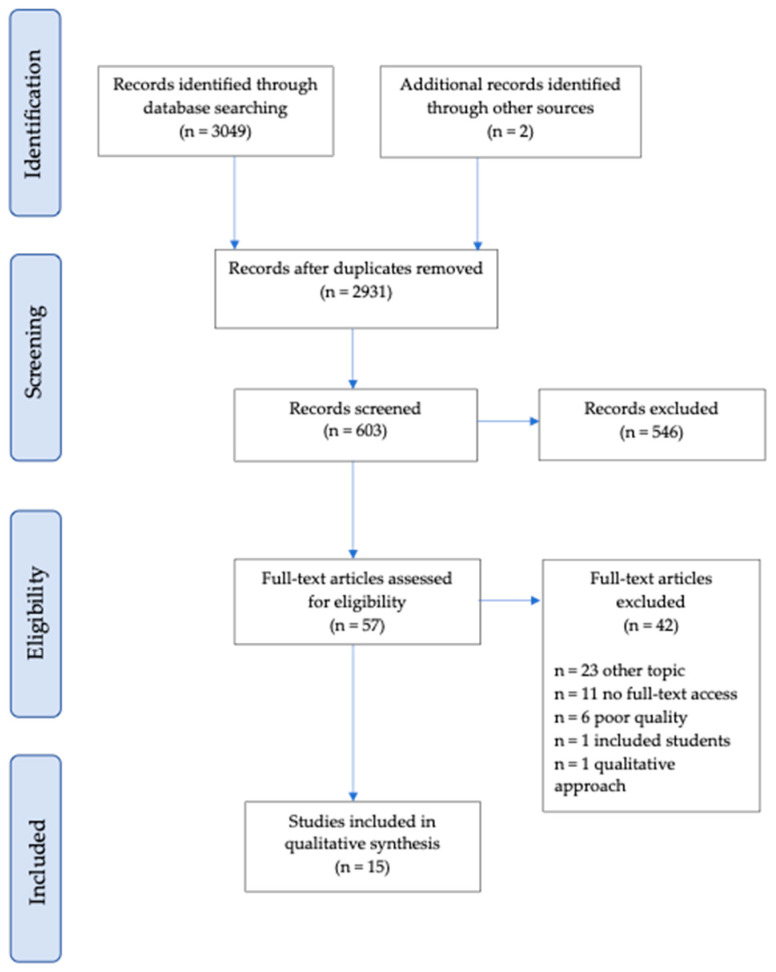
Flow diagram.

A literature search was conducted in four of the most important databases: PubMed, EMBASE, Cinahl, and Scopus (see Figure 1). Key terms were developed considering the population (healthcare workers), context (healthcare services), and objective (discrimination, disparity, and inequality towards the elderly) through a consensus among experts (see Table 1). Additional records were found through a manual search of reference lists of included studies. The last search was conducted on 15 February 2024.

### 3.2. Data Extraction

Articles were imported into the bibliographic manager RefWorks, where duplicates were removed. Two members of the research team independently reviewed the titles and abstracts of the retrieved articles and determined their inclusion based on the inclusion and exclusion criteria.

In the second phase of screening, two authors independently examined the full text of the articles for final inclusion. Any doubts or discrepancies regarding the selection of an article were resolved through discussion, and, when necessary, a third evaluator was involved to make the final decision. To minimize subjectivity in recording the reasons for exclusion, we employed the technique of prioritization and sequential exclusion.

Data were extracted using a predefined tool with a format developed through the consensus of the reviewers. Two members of the team independently extracted the data using this format. Then, data were merged, and any discrepancies were resolved by a third reviewer. The extracted study data included authors, year of publication, geographic region, study design, study population, type of healthcare workers, workplace and personal characteristics, and an ageism scale. Results were extracted in terms of the mean scale score or the prevalence of ageism, when available. All variables studied as potentially influencing levels of ageism were included and described in terms of results (crude and adjusted association analyses). Data were analyzed separately for each type of healthcare worker when available.

### 3.3. Risk-of-Bias Assessment

The quality of the articles was independently evaluated by two authors using the Joanna Briggs Institute Critical Appraisal Tools checklist (JBI) for cross-sectional studies [22]. Each criterion was classified as present or absent, as well as unclear or not applicable. The Joanna Briggs Institute tool evaluates the quality of articles by measuring the representativeness of the source population, sample size, sampling method, study design, use of statistical tests, declaration of conflicts of interest, and declaration of funding sources. To optimize the level of evidence available for this review, only studies of moderate to high quality were included in the analysis. High-quality studies were identified based on a previous meta-analysis [23], which classified studies according to their JBI score, where those scoring above 70% were classified as high quality, between 50% and 69.9% as medium quality, and below 50% as low quality. The results of this evaluation are detailed in Supplementary File S1.

## 4. Results

### 4.1. Participants and Settings

Fifteen studies met the inclusion criteria and are included in this review (see Figure 1). These studies represent a wide range of countries, with nearly half originating from the Middle East (see Table 2). All studies employed a cross-sectional design, except for the study by Afolabi et al. [24], which also incorporated a qualitative approach. The majority of these studies (13 out of 15) aimed to determine ageism in healthcare workers towards older patients and its related factors, while the remaining two focused on ageism as a secondary outcome (see Table 2 for study characteristics and results).

Participants ranged from n = 100 [24] to n = 1367 healthcare workers [28]. Most participants were nurses [24,25,26,27,28,29,30,31,32,33,34,35,36,38] followed by physicians [25,31,32,37], nursing assistants [35], and other healthcare workers such as physiotherapists [32]. The settings varied widely, with most studies being conducted in specialized centers (e.g., hospitals). The majority of participants were young, female, and relatively inexperienced workers (less than 10 years).

### 4.2. Ageism Assessment

Ageism among healthcare workers was assessed using multiple scales, most of which were specific to ageism—the Ageism Attitude Scale (AAS), the Fraboni Scale of Ageism (FSA), the modified version of the Geriatric In-Hospital Nursing Care Questionnaire (GerINCQ), the Kogan’s Older People Scale (KOP), the Relating to Older People Evaluation (ROPE), and the University of California at Los Angeles Geriatrics Attitudes Scale (UCLA-GAS). Others were developed for different purposes—the Geriatric Institutional Assessment Profile (GIAP)—or were questions ad hoc to identify attitudes and barriers toward older age [35]. 

### 4.3. Ageist Perceptions and Attitudes in Health Workers Towards Older Patients

The KOP was the most widely used tool. While most studies reported relatively good scores among their workers [24,28,29], the study by Podhorecka et al. [32] in Poland found neutral mean attitudes in physiotherapists (range 34–204 points). Using the AAS scale (range 23–115 points), Bulut et al. [26] found the best scores in nurses (85 points). Polat et al. [32] found similar results in both nurses and physicians (80.02 and 83.17 points, respectively). However, a study conducted in Ghana by Yakubu et al. [38] reported lower scores in nurses (66.9 points). Another three studies used different versions of the FSA scale to assess ageism. Two of these found neutral attitudes in nurses [27,34]. Other scales also found adequate perceptions and attitudes among nurses using the GerINCQ (45.42 and 56.14 points, respectively) [30], the UCLA-GAS (83.6% positive attitudes) [37] and self-designed questionnaires to assess attitudes and barriers towards the elderly (84.5% positive caring attitude) [35]. Lastly, three studies did not adequately describe their results, even though they aimed to describe ageism among participants [25,36], leaving some participant characteristics unknown [31]. Therefore, although most studies report that healthcare workers generally had a positive caring attitude toward the elderly, their results vary depending on the scale used and the study setting.

### 4.4. Factors Associated with Ageism

To facilitate the synthesis of the results, the various factors analyzed were grouped into three categories: sociodemographic, socioeconomic, and sociocultural factors; personal factors; and work-related factors.

#### 4.4.1. Sociodemographic, Socioeconomic, and Sociocultural Factors

High heterogeneity was observed in the assessment of factors related to ageism. Regarding sociodemographic factors, nine out of fifteen studies analyzed age as a potentially associated factor. However, only two studies (one with multivariate analyses) found a positive association between younger age and negative attitudes toward older people in at least one ageism subscale [25,30]. Less experienced workers were also found to be commonly associated with ageism in these studies, as well as in the studies by Afolabi et al. [24] and Yakubu et al. [38], likely due to the relationship between these variables.

Three out of eleven studies found that male healthcare workers exhibited more ageist attitudes than female workers [30,31,38]. Additionally, the study by Altın and Buran [25] found raw associations between ageist attitudes and being single.

Regarding education-related variables, we must differentiate between three different exposures. First, 10 studies analyzed the relationship between the level of education and ageism within the same category (e.g., bachelor’s degree, master’s degree, specialist training course). These studies showed mixed results, with half indicating lower levels of ageism with higher education levels [19,26,31,34,38], while the other half did not find any relationship [24,27,28,30,32]. Second, four studies examined this relationship, but compared different professional categories. In this regard, two studies found that physicians showed less ageism than nurses [31,32], while another study found opposite results (nurses showed less ageism than physicians) [25]. For its part, the study by Salia et al. [35] found that nurses showed less ageism than nursing assistants. However, only the study by Altın and Buran [25] appropriately described the differences in specific geriatric training across professional profiles. Lastly, nine studies addressed this relationship between education and ageism but regarding specific geriatric training courses or aging knowledge. This question was addressed mainly using the Palmore’s Facts on Aging Quiz (FAQ) [24,28,29,34] or ad hoc questions [25,27,30,35,37]. Most of the studies using the FAQ found a relationship between better aging knowledge and fewer ageist attitudes, with the exception of the study by Afolabi et al. [24] in nurses. However, studies using ad hoc questions found inconsistent results.

Other social-related factors, such as religion, ethnicity, or economic status, were less frequently explored and did not appear to have a strong relationship with ageism levels.

#### 4.4.2. Personal Factors

The most extensively explored factor was family experience and/or the quality and quantity of contact with older adults. This variable was primarily measured using ad hoc questions or the Contact with Elderly People Scale (CEP) [27]. Many studies found a relationship between contact frequency or experience with older people and the presence of ageist perceptions or attitudes [27,28,30,31,32]. However, some studies did not find this relationship, likely due to the heterogeneity in how this aspect was measured.

Three studies explored the relationship between personality characteristics and ageism. They found that empathy [32] and anxiety about aging (using the Anxiety about Aging Scale (AnAS)) [27,29] were associated with ageist attitudes.

Another variable that was generally associated with ageism was the desire to work with older people. Although it was only assessed in six studies, four of these found a significant association [26,28,29,31]. 

#### 4.4.3. Work-Related Factors

Most of the studies included workplace factors in their investigations, primarily aiming to compare situations across different settings.

Regarding various hospitals and units, it is worth noting that only three studies assessed differences between units. These studies found that ER nurses [25] and healthcare workers who had not worked in geriatric units exhibited higher levels of ageism [24,33,39]. Furthermore, two studies conducted in the Middle East also identified differences based on the type of hospital, with public hospitals being rated the worst [31,34].

Workload was not explored in any of the studies. However, other variables related to workload, such as shift work, were not associated with ageism [30]. Additionally, the study by Bulut et al. [26] investigated self-reported factors associated with ageism. The most significant factors identified were lack of personnel, job fatigue, stress, and working conditions.

#### 4.4.4. Predictive Factors of Ageism

Predictive factors for the presence of ageism were as follows (see Table 2 for details): younger workers [30], less educated [34,35], less experienced in work [24,30] and less experienced in elder relationships [28,30,32]. Additionally, those who did not wish to work with older people [28,29,40], those who showed less empathy [32] and those who exhibited anxiety about aging [27] were also predictive factors for the presence of ageism.

#### 4.4.5. Study Quality

Only four studies were determined to be of high quality (≥6 out of 8 items), whereas the remaining were found to be of moderate quality (4 to 6 items).

The vast majority of the studies described their participants and settings in detail. All participants were healthcare workers exposed to geriatric care. Although many types of healthcare workers were analyzed, most of them exhibited similar sociodemographic characteristics, facilitating comparison between studies. However, some biases were detected during the review process. For example, the study by Salia et al. [35], compared nurses (n = 134) versus nurse assistants (n = 8), but the representativeness of nurse assistants may be compromised.

In relation to this, some studies were found to report high levels of rejection among their participants [36] or did not provide details about the final sample [24,25,32,33,34]. This could introduce sampling bias.

The study by Salia et al. [35] used ad hoc questions to evaluate ageism among their workers; however, its primary aim was to determine which factors affect the care of elderly patients. Additionally, many other variables were also evaluated ad hoc, such as aging education or knowledge, which may lead to erroneous associations.

Most of the studies did not identify all potential confounding factors related to ageism. In fact, only the studies by Altın and Buran [25] and Polat et al. [33] reported this information comprehensively. However, both studies provided poor analysis to properly determine which factors were related to the presence of ageism.

Lastly, only eight studies used multivariate analysis to explore the association of the described variables with ageism levels [24,27,28,29,30,32,34,35]. Nevertheless, Rababa et al. [34] did not include education level in their model, even though it was statistically associated with ageism levels assessed by FSA.

## 5. Discussion

The primary objective of our review was to assess the presence of ageism among healthcare professionals and to identify the factors associated with its presence.

Generally, relatively low levels of ageism were found in our review among healthcare professionals, although these results varied across studies. The heterogeneity of results may be related to the different cultural contexts in which they were conducted. Almost half of the studies in our review were conducted in the Middle East; therefore, we acknowledge that there is limited understanding of the situation of ageism in other continents, where sociocultural factors may play a significant role. In cultures that value the elderly and promote their integration into family and community life, such as many Asian societies, the degree of ageism tends to be lower [41]. Conversely, in societies with social structures that foster the segregation of the elderly, such as nursing homes rather than multigenerational living arrangements, a higher degree of ageism may be experienced [42]. Despite the potential impact of cultural context on the level of ageism within a population, factors related to society, such as religion, ethnic origin, or economic status, were less explored in our review. This underscores the need for further exploration of the influence of cultural context on the development of ageism. Future studies should investigate the situation in other countries around the world and whether cultural context also influences negative attitudes towards older adults within the healthcare population.

Regarding the other sociodemographic variables explored, age and gender did not show any significant relationship with ageism in our review. Another review of ageism in nurses also found inconsistent results concerning the demographic characteristics of nurses and the presence of ageism [43]. Additionally, regarding education-related variables, our review found mixed results regarding the level of education in general, as well as among different professional categories. This is likely due both to the differences in how these variables are addressed across studies and to the varying curricula for each profession in different countries, which differ significantly. However, a significant relationship was identified between aging knowledge (assessed by the FAQ) and ageism among healthcare workers [28,29,34]. The available literature on nurses aligns with these findings [43], indicating that advanced geriatric training can foster a deeper understanding and recognition of aging issues even among undergraduate students across various disciplines [44,45]. Therefore, specific training in geriatrics is crucial to ensure the well-being and quality of life of our elderly population [46]. 

In terms of overall professional experience, our review did not identify any strong associations with ageism scores. This finding may be influenced by the relatively young age of the participants in most of the included studies. Limited evidence was found suggesting that related variables, such as experience in geriatric units [24,33], family experience, or contact with older adults [27,28,30,31,32], were associated with lower levels of ageism. However, not all studies analyzing these relationships reported positive results. This inconsistency may stem from differences in cultural contexts and the varied approaches used to measure personal and professional experience with older adults, with only one study employing a validated scale to assess these variables [27]. Such experiences may contribute to reducing stereotypical beliefs and discriminatory behaviors by fostering greater understanding and empathy toward older adults [47]. On the other hand, individuals expressing interest and a desire to work with older adults may inherently exhibit fewer ageist beliefs and behaviors [48,49]. This trend was also observed in our review among those expressing a willingness to work with older patients [26,28,29,31]. 

Other personal factors, such as sexism and anxiety about aging, may also be linked to ageism in healthcare professionals, just as they are in students [50]. Similarly, age discrimination in the healthcare system could be exacerbated by issues of ethnicity or racism [51]. However, in our review, only two articles explored the association between ageism and anxiety about aging, both reporting positive results [27,29]. Other factors, such as sexism or racism, remain unexplored. Therefore, future studies should determine whether sexism and racism coexist with ageism among healthcare professionals.

Lastly, job-related factors such as workload or understaffing could be associated with ageism among healthcare professionals, although only one study has considered this variable subjectively in its analyses [26]. Indeed, a qualitative systematic review describes healthcare for older adults as a significant workload for nurses [20]. Consequently, future research should investigate the relationship between socioeconomic conditions, work environments (e.g., hospital versus primary care), and individual job satisfaction with ageism among healthcare professionals, issues that remain largely unaddressed to date.

### 5.1. Practical Implications

Healthcare institutions must recognize that the problem of ageism among their professionals is a reality and conduct studies aimed at determining its presence. These studies must investigate both personal (internal) and cultural and job-related (external) factors associated with its presence, which may negatively impact the care provided to older patients. This evidence should serve as a foundation for developing targeted strategies to address this issue. Approaches based on geriatric education, contact with older adults, or a combination of both are considered effective in reducing prejudice and negative attitudes toward older adults in the general population [52] and among students in health and social sciences [53,54]. Consequently, it is reasonable to expect that educational programs could also be effective for licensed professionals. The ultimate goal, therefore, should be to ensure interactions free from stereotypes, prejudices, and discrimination against older patients.

### 5.2. Study Limitations

The present review has several limitations.

First, it is important to note that our review aimed to identify ageism and related factors across a diverse range of healthcare workers. Due to the design of the studies, it has not always been possible to differentiate the results based on the type of healthcare worker. Consequently, the participants, aims, and settings varied significantly from one study to another. However, this review provides, for the first time, a general overview of this phenomenon among the population studied.

Second, and with regard to ageism, the literature identifies three dimensions: stereotypes, prejudices, and discrimination. According to a recent review, not all scales assess all of these dimensions [55]. Although the Kogan’s Attitudes Towards Older People Scale (KOP) is the most commonly used in this review [56], it only assesses stereotypes and prejudices. In contrast, only three studies utilized the Fraboni Scale of Ageism [57], which is designed to assess all three dimensions. Additionally, the study by Salia et al. [35] did not use a validated scale to assess ageism. This limitation may impact the results reported here by potentially underestimating the magnitude of the problem. Similarly, sociodemographic, socioeconomic, personal, and workplace factors potentially associated with ageism vary widely across studies. The fact that nearly half of the studies in this review were conducted in the Middle East may lead to a limited understanding of the situation in other parts of the world, where social and cultural characteristics could differ. Moreover, some studies did not present robust analyses to confirm the presence of associations, limiting the generalizability of the findings.

Lastly, although studies were of moderate-to-high quality (as assessed by the JBI), some did not report their data adequately (in terms of inclusion and exclusion criteria, participants’ characteristics, healthcare type, and/or ageism scores). Furthermore, the majority of the studies were conducted with nurses. Therefore, the healthcare workers represented here may not fully reflect the broader reality of health professionals. Nonetheless, low rates of ageism were found among health care professionals, as was the association of ageism with these professionals’ education and experience of aging. This aligns with the general population and thus gives reliability to this review. Further research should compare different types of professionals and address all potential confounders to better understand this phenomenon.

## 6. Conclusions

Healthcare workers generally had a positive caring attitude toward elderly patients, though the literature on this topic remains scarce and the results could vary depending on the cultural context. The main factors associated with ageism were specialized training in geriatrics and the years of experience in geriatric units. Other factors, such as intergenerational contact and a desire to work with older patients, may also be linked to a lower presence of ageism among healthcare professionals. Further studies are needed to confirm these results, along with the development of interventions aimed at providing respectful and equitable healthcare.

## Figures and Tables

**Table 1:** Search strategies in each of the databases.

Medline (PubMed)	((“Ageism”[Title/Abstract] OR “Ageist”[Title/Abstract] OR “Aged”[Title/Abstract] OR “elder*”[Title/Abstract] OR “Aging”[Title/Abstract] OR “Older”[Title/Abstract] OR “Senior”[Title/Abstract] OR “Ageism”[MeSH Terms]) AND ((“Discrimination”[Title/Abstract] OR “Disparities”[Title/Abstract] OR “Stigma”[Title/Abstract] OR “stereoty*”[Title/Abstract] OR “Prejudice”[Title/Abstract] OR “attitude*”[Title/Abstract] OR “Ageism”[Title/Abstract] OR “Ageist”[Title/Abstract]) AND ((“Health care personnel”[Title/Abstract] OR “Health care staff”[Title/Abstract] OR “health care provider*”[Title/Abstract] OR “Health care”[Title/Abstract] OR “Health services”[Title/Abstract] OR “health occupations”[MeSH Terms]))
Scopus	ABS/TIT/KEY (ageism OR ageist OR aged OR elder* OR aging OR older OR senior) AND ABS/TIT/KEY (discrimination OR disparities OR stigma* OR stereoty* OR prejudice OR attitude* OR ageism OR ageist) AND ABS/TIT/KEY (“Health care personnel” OR “Health care staff” OR “health care provider” OR “health care” OR “health services”)
EMBASE	(ageism:ab,ti OR ageist:ab,ti OR aged:ab,ti OR elder*:ab,ti OR aging:ab,ti OR older:ab,ti OR senior:ab,ti) AND (discrimination:ab,ti OR disparities:ab,ti OR stigma*:ab,ti OR stereoty*:ab,ti OR prejudice:ab,ti OR attitude*:ab,ti OR ageism:ab,ti OR ageist:ab,ti) AND (‘health care personnel’:ab,ti OR ‘health care staff’:ab,ti OR ‘health care provider*’:ab,ti OR ‘health care’:ab,ti OR ‘health services’:ab,ti)
Cinahl	TI (Ageism OR Ageist OR Aged OR elder* OR Aging OR Older OR Senior) AND TI (Discrimination OR Disparities OR Stigma* OR stereoty* OR Prejudice OR attitude* OR Ageism OR Ageist) AND TI (“Health care personnel” OR “Health care staff” OR “health care provider*” OR “Health care” OR “Health services”)

**Table 2:** Study characteristics and results (n = 15).

Author (Year) Country	Study Design	Sample	Sample Characteristics	Scales	Results
Afolabi et al. (2020) [24] Nigeria	Mixed Aim: to assess nurses’ knowledge and attitudes toward the care of elderly patients with dementia and explore associated factors	n = 100 Nurses and midwives in one hospital	50% female 54% 30–39 years 82% married 93% Christians 38% bachelor degree 72% < 10 years work experience 71% no previous elderly care experience	Ageism: KOP Aging knowledge: FAQ	The majority of the nurses have good knowledge and attitudes toward the care of elderly patients. No factors affected nurses’ FAQ (all *p*’s > 0.05). No previous experience in special care unit for elderly was associated with worse attitudes (KOP) (*p* < 0.001). Adjusted analysis showed an elevated aOR = 2.90 (*p* < 0.05) for this parameter.
Altın and Buran (2022) [25] Turkey	Cross-sectional Aim: to investigate the attitudes of physicians and nurses about ageism in the COVID-19 pandemic	n = 308 Physicians (n = 154) and nurses (n = 154) from different units in two hospitals	58.1% > 35 years 76.6% women 62.7% married 57.5% living with elderly relatives 23.7% preferred young patients 20.1% geriatrics education (56% of these were nurses)	Ageism: UCLA-GAS Subscales: - Social values - Medical care - Compassion - Resource distribution	Physicians preferred young patients when compared with nurses (*p* < 0.001). Younger and less experienced workers showed more ageism attitudes assessed by UCLA-GAS and its subscales (all *p*’s < 0.01). Married workers showed less UCLA-GAS and compassion and medical care subscales (*p*’s < 0.01). Nurses showed better resource distribution subscale scores than physicians (*p* < 0.01). Different units showed different ageism levels (*p* < 0.01), with worse levels found in ER.
Bulut et al. (2016) [26] Turkey	Cross-sectional Aim: to determine surgical nurses’ attitudes towards the elderly	n = 337 Surgical nurses in seven different hospitals	64.7% 18–35 years 87.5% female 61.5% married 57% post-graduate degree 79% less than 11 working years	Ageism: AAS Subscales: - Restricting the elderly: AAS-RE - Positive: AAS-PA - Negative: AAS-NA Self-reported ageism related factors (lack of personnel, job fatigue and stress, unsuitable working conditions, lack of knowledge, cultural and religious factors, prejudice)	AAS: 85.0 ± 7.7 AAS-RE: 36.5 ± 3.7 AAS-PA: 30.4 ± 4.4 AAS-NA: 18.0 ± 3.1 Self-reported ageism related factors: lack of personnel (82.2%), job fatigue (77.2%), stress (68.5%), and working conditions (56.4%). Ageism decreased with educational level (AAS, AAS-RE and AAS-NA *p* < 0.001), though this was not significant for AAS-PA. Ageism decreased with “wish to work with aged people” (AAS, AAS-RE and AAS-PA *p* < 0.001), though this was not significant for AAS-NA.
Hwang and Kim (2021) [27] South Korea	Cross-sectional Aim: to identify factors influencing nurses’ ageism attitudes	n = 162 Nurses (17.3%) and nurse managers (82.7%) in two general hospitals	35.36 ± 8.84 years 100% females 51.9% married 74.7% bachelor degree 58% religious 10.37 ± 8.96 work experience years 93.2% geriatric work experience 91.4% did not cohabite with older adults, while 54.9% had in the past 71% gerontological education	Ageism: FSA Subscales: - Emotional avoidance: FSA-EA - Discrimination: FSA-D - Stereotype: FSA-S Emotional intelligence: WLEIS Experience with older people: frequency (CEP-CF) and quality (CEP-CQ) Anxiety about aging: fear of old people (AnAS-FOP), psychological concerns (AnAS-PC), physical appearance (AnAS-PA) and fear of loss (AnAS-FL)	FSA 2.6 ± 0.36 FSA-EA 2.6 ± 0.50 FSA-D 2.5 ± 0.40 FSA-S 2.7 ± 0.45 WLEIS 3.4 ± 0.42 CEP 4.0 ± 1.15 AnAS 2.9 ± 0.42 FSA was associated with cohabitation with an old adult in the past (*p* < 0.05). FSA correlated with CEP (r = −0.28) and its subscales (*p* < 0.001) and AnAS (r = 0.37) and its subscales (*p* < 0.001), with the exception of AnAS-PC; WLEIS did not correlate. Factors influencing FSA were AnAS-FOP (β = 0.34) and AnAS-FL (β = 0.28) (*p*’s < 0.001).
Lan et al. (2019) [28] China	Cross-sectional Aim: to assess the attitude of nurses toward the care of older adults	n = 1367 Nurses of five tertiary hospitals	30.9 ± 7.08 years 97.5% female Junior college 48.9% 47.2% < 5 years work experience 81.5% geriatrics experienced 80.4% live with an older adult 62.3% were cared for by older adults 60.2% self-referred a good relationship with older adults	Ageism: KOP Aging knowledge: FAQ	KOP 155.1 ± 21.94 FAQ 7.1 ± 4.36 Non-experienced nurses, those who self-referred as not having good relationships with older adults, those who reported not having been cared by older adults (*p* < 0.001), low FAQ (*p* < 0.01), and those who did not live with older adults (*p* < 0.05) showed worse KOP scores. Those who reported a bad relationship with older adults (aOR = 2.44; 95% CI: 1.81–3.29) and those who have not cared for older adults (aOR = 1.55; 95% CI: 1.13–2.11) remained significant after adjusting for confusing factors.
Liu et al. (2014) [29] UK	Cross-sectional Aim: to establish an explanatory model of nurses’ attitudes towards older people and working with older patients	n = 579 Nurses attending professional education courses within a large university	36.9% 30–39 years 89.6% female 89.8% did not live with an older person 81.2% worked in a hospital 9.6 ± 8.21 work experience years 49.2% bachelor’s degree 32.4% surgical wards 57.2% staff nurses	Ageism: KOP Aging knowledge: FAQ Anxiety about aging: AnAS Attitudes towards healthcare resources allocation scale Wish to work with older people	KOP 170.7 ± 18.88 Worse KOP scores were associated with worse AnAS scores, attitudes towards healthcare resources allocation, black background (*p* < 0.001), staff nurses, and less FAQ (*p* < 0.05). Regression analysis for the desire to work with older people showed a significant association with ageism (KOP) (aOR = 1.03; 95% CI: 1.01–1.05; *p* < 0.01).
Modarres Sadraei et al. (2022) [30] Iran	Cross-sectional Aim: to identify the perception and attitude of nurses toward elderly care and their correlation with professional responsibility in nurses	n = 252 ER nurses in eight different hospitals	31.8 ± 6.27 years 81% female 8.4 ± 5.74 work experience years 4.1 ± 3.03 ER experience years (61.2% only ER experience) 92.8% clinical nurse 74.1% shift workers 86.7% bachelor’s degree 90.1% did not receive geriatrics course 86.1% live with an elderly person	Ageism: GerINCQ Subscales: - Perception - Attitude - Professional responsibility	45.42 ± 7.53 for perception Nurses’ perceptions were not associated with any sociodemographic characteristic. 56.14 ± 12.35 for attitude Attitudes were associated with living with the elderly (*p* < 0.01) and gender (*p* < 0.05). 39.62 ± 9.86 for responsibility Professional responsibility was associated with gender, living with the elderly, age and job experience (*p* < 0.05). Regression model for professional responsibility found that perception (β = 0.08), attitude (β = 0.06), job experience (β = 0.04), age (β = 0.02) (*p* < 0.001), and living with the elderly (β = 0.04) were predictors for ageism (*p* < 0.01).
Ozel Bilim and Kutlu (2021) [31] Turkey	Cross-sectional Aim: to determine the psychometric properties, factor analysis, and cut-off value for the FSA in a sampling of healthcare workers	n = 814 Nurses (n = 545) and physicians (n = 269) in state and university hospitals	38.0 ± 9.12 years 26.7% female 177.6 ± 43.50 working hours per month 15.4 ± 9.57 working years 38.2% bachelor’s degree 65.8% state hospitals 67% nurses 62% worked on call 75.1% satisfied with work	Ageism: FSA	Women, less educated, nurses, employees at state hospitals, employees unsatisfied with their jobs (*p* < 0.001), those who live in nuclear families, and employees working on call (*p* < 0.01) showed worse FSA scores.
Podhorecka et al. (2022) [32] Poland	Cross-sectional Aim: to assess the approach of Polish physiotherapists to the elderly and to analyze the factors influencing it	n = 252 Physiotherapist in Poland’s National Chamber of Physiotherapists	75% female 41.67% 30–39 years 55.16% married 76.19% master’s degree 35.71% < 5 years work experience 40.87% working at home 65.87% living with an older person	Ageism: KOP Empathy: JSE	KOP 100.7 ± 17.46 JSE 106.31 ± 12.38 82.54% self-referred personal and 86.51% professional contact with older people. 59.13% self-reported that keeping contact with older people is important. JSE (β = 0.12) and relevance of contacts with elderly people (β = 1.24) were associated with KOP punctuation.
Polat et al. (2014) [33] Turkey	Cross-sectional Aim: to determine the perceptions of elderliness and the prevalence of ageism among nurses and physicians	n = 167 Nurses (n = 110) and physicians (n = 57) in medical and surgical units of a university hospital	Nurses/physicians 52.7%/49.1% 28–35 years 94.5%/63.2% female 53.6%/28.1% married 94.5%/100% undergraduate 50.9%/59.6% lived with an older person 60.9%/15.8% > 5 years work experience 51.8%/68.4% medical units 92.7%/77.2% currently working in older patient units 68.2%/64.9% satisfied by serving older people 71.8%/–did not receive geriatrics course	Ageism: AAS Subscales: AAS-NA AAS-PA AAS-RE	Nurses AAS 80.02 ± 7.64 Physicians AAS 83.17 ± 9.09 Physicians showed less ageism than nurses when measured by AAS, and AAS-RE (*p* < 0.05). Previous work in older patients units was associated with less ageism in nurses (only for AAS-RE) and physicians (only for AAS-NA) (*p* < 0.05).
Rababa et al. (2020a) [34] Jordan	Cross-sectional Aim: to examine how the sociodemographic and professional characteristics of nurses correlate with their levels of knowledge, attitudes, and ageism toward older adults.	N = 317 Nurses in medical, surgical, ER, and ICU in two different hospitals	31.1 ± 4.75 years 44.2% female 60.9% bachelor’s degree 7.4 ± 4.39 years of work experience 50.6% living with an elderly person	Ageism: ROPE-Negative ageism Ageism: FSA Aging knowledge: FAQ1	ROPE 19.1 ± 4.9 FSA 69.5 ± 13.8 FAQ1 11.8 ± 3.3, higher in married nurses and ICU (*p* < 0.01). Lower education level (*p* < 0.01) and public hospitals (*p* < 0.05) were associated with worse ROPE, and FSA outcomes. Multivariate analysis revealed that negative ageism (ROPE) was only associated with FAQ1 (β = −0.178) and FSA (β = 0.517) (*p* < 0.001) but education level was not included in the model.
Salia et al. (2022) [35] Ghana	Cross-sectional Aim: to assess factors affecting the care of elderly patients among nursing staff	n = 142 Nurses (n = 134) and nurse assistants (n = 8) in several units at a tertiary hospital	38.7% 31–40 years 70.4% female 51.4% married 50% had at least master’s degree 12% were geriatrics specialized nurses 45.8% 6–10 years work experience	Ageism: Self-designed questionnaire which included attitude and barriers towards older age Self-designed questionnaire which included aging knowledge, characteristics or facilities	84.5% had a positive caring attitude towards the elderly and 88.7% good knowledge in elderly care. Nurse assistants showed less positive caring attitudes (more ageism than nurses) (aOR = 0.16, *p* < 0.05). Good aging knowledge (aOR = 4.13, *p* < 0.05) and nursing managers showed more positive attitudes towards older age (aOR = 6.80, *p* < 0.05).
Tavares et al. (2017) [36] Portugal	Cross-sectional Aim: to analyze the relationship between the perceptions of nurses about the geriatric care environment and geriatric nurses’ knowledge and attitudes according to unit type and regions	n = 1068 Nurses in medical units, surgical units, and ICU/ER in five hospitals	79.7% female 34.1 ± 8.5 years 45.4% single 11.3 ± 8.4 years of work experience 10 ± 8.1 years of work experience in the current institution 7.5 ± 6.5 years of work experience in the current unit 88.8% degree in nursing 86.3% not gerontological education	Ageism: GIAP Subscales: - Geriatric nursing knowledge/attitudes scale - Geriatric care environment - Professional issues	No differences were found in geriatric nursing knowledge and attitudes subscale. The geriatric care environment subscale was different only between north and central ICU/ER nurses (north showed better scores) (*p* < 0.05). The professional issues subscale was different only between north and central medical units nurses (central showed better scores) (*p* < 0.05).
Tufan et al. (2015) [37] Turkey	Cross-sectional Aim: to investigate the attitudes of internal medicine residents toward older people	n = 274 Internal medicine residents in six public university hospitals	50.3% female 28 ± 2 years 2 ± 2.58 years of work experience 53.6% did not receive gerontological education 39.1% made a rotation in geriatrics during residence 90.1% had elderly relatives 8.4% lived with elderly relatives	Ageism: UCLA-GAS	83.6% had positive attitudes toward older people. Undertaking of a geriatrics course was associated with positive attitudes (*p* < 0.05). Geriatrics rotation during residency arose as a protector factor for ageism (cOR = 2.6; *p* < 0.05).
Yakubu et al. (2022) [38] Ghana	Cross-sectional Aim: to evaluate nurses’ attitudes towards older adults	n = 160 Nurses in a tertiary hospital in any of the medical and surgical wards	30.1 ± 3.8 years 58.1% female 4.4 ± 3.3 years of work experience 52.5% diploma nurses 48.1% Christian	Ageism: AAS Subscales: - Restricting the elderly: AAS-RE - Positive: AAS-PA - Negative: AAS-NA	AAS: 66.9 ± 6.05 AAS-RE: 30.59 ± 3.7 AAS-PA: 24.26 ± 3.3 AAS-NA: 12.16 ± 2.4 More educated nurses showed less ageism in the AAS-RE (r = −0.296; *p* < 0.01) and AAS-NA (r = −0.161; *p* < 0.05) subscales. Experienced nurses showed less ageism than less experienced nurses (r = 0.672; *p* < 0.05). Female nurses showed less ageism than male counterparts (*p* < 0.05).

AAS: Ageism Attitude Scale; AAS-NA: AAS subscale negative ageism; AAS-PA: AAS subscale positive ageism; AAS-RE: AAS subscale restricting the life of the elderly; AnAS: Anxiety about Aging Scale; AnAS-FL: AnAS subscale fear of loss; AnAS-FOP: AnAS subscale fear of old people; AnAS-PA: AnAS subscale physical appearance; AnAS-PC: AnAS subscale psychological concerns; aOR: adjusted odds ratio; CEP: Contact with Elderly People; CEP-CF: CEP subscale contact frequency; CEP-CQ: CEP subscale contact quality; cOR = crude odds ratio; ER: emergency room; FAQ: Palmore’s Facts on Aging Quiz; FSA: Fraboni Scale of Ageism; FSA-D: FSA subscale discrimination; FSA-EA: FSA subscale emotional avoidance; FSA-S: FSA subscale stereotype; GerINCQ: The modified version of the Geriatric In-Hospital Nursing Care Questionnaire; GIAP: Geriatric Institutional Assessment Profile; JSE: the Jefferson Empathy Scale; ICU: intensive care unit; KOP: Kogan’s Older People Scale; ROPE: Relating to Older People Evaluation; UCLA-GAS: University of California at Los Angeles Geriatrics Attitudes Scale; WLEIS: The Wong and Law Emotional Intelligence Scale.

## Data Availability

No new data were created in this study. Data sharing is not applicable to this article.

## Supplementary Materials

The following supporting information can be downloaded at https://www.mdpi.com/article/10.3390/nursrep14040295/s1, File S1: Study quality assessed by the JBI cross-sectional checklist (n = 15).

## Public Involvement Statement

No public involvement in any aspect of this research.

## Guidelines and Standards Statement

This study was guided by the Preferred Reporting Items for Systematic Reviews and Meta-Analyses (PRISMA) [21].
