# Supplementary material for: Ageism and Associated Factors in Healthcare Workers: A Systematic Review
Source: Nurs Rep. 2024 Dec 16;14(4):4039–59. doi: 10.3390/nursrep14040295 (PMC11677396; doi:10.3390/nursrep14040295)
Supplement: Supplementary file 1 [file nursrep-14-00295-s001.zip › nursrep-3221786-supplementary.pdf]

Supplementary File S1. Study quality assessed by the JBI cross-sectional checklist (n = 15).

Author (Year)	Q1	Q2	Q3	Q4	Q5	Q6	Q7	Q8	Total
Afolabi et al. (2020) [24]	N	U	Y	Y	N	Y	Y	Y	5/8
Altın & Buran (2022) [25]	Y	Y	Y	Y	Y	Y	Y	N	7/8
Bulut et al. (2016) [26]	Y	Y	U	Y	N	Y	Y	N	5/8
Hwang & Kim (2021) [27]	Y	Y	Y	Y	N	Y	Y	Y	7/8
Lan et al. (2019) [28]	Y	U	Y	Y	N	N	Y	Y	5/8
Liu et al. (2014) [29]	U	Y	Y	Y	N	N	Y	N	4/8
Modarres Sadraei et al. (2022) [30]	Y	Y	Y	Y	N	U	Y	U	5/8
Ozel Bilim & Kutlu (2021) [31]	N	Y	Y	Y	Y	N	Y	Y	6/8
Podhorecka et al. (2022) [32]	N	N	U	Y	N	Y	Y	Y	4/8
Polat et al. (2014) [33]	Y	U	Y	Y	Y	N	Y	U	5/8
Rababa et al. (2020a) [34]	Y	Y	Y	Y	N	N	Y	N	5/8
Salia et al. (2022) [35]	Y	Y	Y	Y	N	Y	Y	Y	7/8
Tavares et al. (2017) [36]	Y	Y	Y	Y	N	N	Y	N	5/8
Tufan et al. (2015) [37]	N	Y	Y	Y	N	Y	Y	N	5/8
Yakubu et al. (2022) [38]	Y	Y	U	Y	N	U	Y	N	4/8

N: No; U: Unclear; Y: Yes.
